# The impact of food stimuli and fasting on cognitive control in task switching

**DOI:** 10.1007/s00426-023-01884-y

**Published:** 2023-10-19

**Authors:** Viktoria Maydych, Hanna Pöschel, Sebastian Kübler, Torsten Schubert

**Affiliations:** 1 Institute for Psychology, Martin Luther University Halle-Wittenberg, Emil-Abderhalden-Str. 26-27, 06108 Halle (Saale), Germany; 2 Department of Psychology, Humboldt-University, Berlin, Germany

## Abstract

Previous research demonstrated motivation-control interactions in task switching. However, motivational effects on switch costs have been mostly examined using monetary rewards. Here, we investigated whether stimulus material linked to food and fasting affect control processes in task switching. We predicted that switching to the task comprising food stimuli would be facilitated, which should result in lower switch costs for this task, and that these effects would be stronger with higher motivational salience of the food stimuli, i.e. in hungry individuals and/or individuals with restrictive eating. Participants switched between categorising food items as sweet or savoury and digits as odd or even in two task-switching paradigms: an alternating runs and a voluntary task switching. Hunger was induced by 14 h fasting in the experimental compared to the control group. Results showed lower switch costs for the motivational-affective food task in both task-switching paradigms and in both groups. Switch costs for the neutral digit task were significantly higher in the fasting group compared to the control group in alternating runs task switching only. Individual differences in restrictive eating were related negatively but not significantly to the size of the switch costs. All in all, the results demonstrate an impact of motivational-affective stimuli on cognitive control in task switching and suggest a potential modulatory role of motivational states, though the findings need to be replicated.

## Introduction

In daily life, people often perform different tasks at the same time or in close succession. In these situations, individuals flexibly switch from one task to another, while responding to external demands and internal goals. In the last decades, research in task switching has mainly focused on the investigation of cognitive control processes, which underlie cognitive flexibility. Although this research has been very fruitful to define control mechanisms recruited to perform and to switch between tasks, the emotional and motivational aspects of task switching have been largely neglected. Since most of daily multitasking situations take place in emotional and/or motivational context, the relevance of these factors needs stronger recognition in task-switching research. Indeed, increasing evidence from behavioural and imaging studies suggests that cognitive control processes are influenced by emotional and motivational states, highlighting the need to investigate emotion-motivation-control interactions within the task-switching approach in more detail (for reviews see Botvinick & Braver, 2015; Yee & Braver, 2018). In the current study, we addressed this issue by examining whether a manipulation of the motivational salience of one of the tasks and the motivational state of participants affect cognitive control in task switching.

### Task switching

In task-switching situations, participants usually switch between two tasks in quick succession. Depending on the particular paradigm, the task to be performed on a given trial can be indicated by the position of the task in a predictable task sequence (alternating runs; Rogers & Monsell, 1995), externally cued (cued task switching; Meiran, 1996), or selected voluntarily (voluntary task switching; Arrington & Logan, 2005). In alternating runs paradigm, participants usually perform two tasks that change in predictable manner every n*th* trial. The number of trials before change (runs) remains constant for a block or for the whole experiment (e.g., resulting in AABBAABB order of tasks A and B). That is, participants are informed which task to perform by the position of this task within the run or the relevant task is additionally indicated by a special task cue (e.g., stimulus position, colour, words, etc.). In contrast, in voluntary task switching, participants switch between two tasks without the current task being signaled by any kind of external cue. Instead, participants can freely decide which task to perform on a given trial although in some studies additional effort is provided in order to prevent at least systematic strategies and to reduce the risk for systematic choice biases (see Arrington & Logan, 2004, and below).

Robust findings for these paradigms are longer reaction times (RTs) and higher error rates on task switch trials compared to task repetition trials, a performance decrement termed as *switch costs*. In voluntary task switching, which allows for assessment of task selection as an additional performance measure, a further key finding is a tendency of participants towards repeating tasks more often than would be expected by chance, the *repetition bias*. Although participants usually show no bias towards any of the tasks, the commonly used instruction to perform the tasks in random order is often not exactly followed by participants (Arrington & Logan, 2004, 2005; Mittelstädt et al., 2018).

Depending on the theoretical view, the cause for the switch costs is explained by assuming active top-down and/or passive bottom-up processes. According to *top-down* accounts, switch costs reflect executive control processes of reconfiguring the task set on switch trials. That is, switching requires activation of the relevant task set, i.e. activation of a task goal, remembering the task rules, and stimulus–response mappings, and inhibiting the task set that is no longer relevant (Mayr & Kliegl, 2000). As an alternative, *bottom-up* approaches suggest that *switch costs* reflect rather passive processes related to interference from the prior task set. In this case, interference is explained in terms of incomplete deactivation of the previous task and persistent suppression of the task of the current trial, which causes additional processing costs (Allport et al., 1994). While many studies on task switching are concerned with the specification of these mechanisms, the present research focuses on the question of how motivation interacts with cognitive control and related costs in task-switching situations.

### Task switching and motivation

The impact of motivation on task switching can have different facets. Many researchers assume that motivation enhances cognitive control in task processing, e.g. by boosting active maintenance of task goals and components in working memory (Braver et al., 2014), increased perceptual and attentional processing (Calvo & Lang, 2004; Spaniol et al., 2011), improved memory encoding (Adcock et al, 2006), etc. On the other side, motivational impact can also be harmful to task processing, e.g. when motivationally salient but no longer goal-relevant stimuli are disturbing task performance (Ward et al., 2019).

Related evidence has been reported by studies that manipulated the reward value of tasks using monetary incentives and measured performance when switching between rewarded and non-rewarded tasks. Such studies demonstrated enhanced control over the representation of the rewarded task, which resulted in lower switch costs when switching to this task (Kleinsorge & Rinkenauer, 2012; Shen & Chun, 2011; Umemoto & Holroyd, 2015). However, on the opposite, once a rewarded task had been activated, the deactivation of that task became more challenging, particularly when switching to the non-rewarded task (Jiang & Xu, 2014).

An alternative approach for the investigation of a potential affect-motivation-control interaction in task switching is to examine how personal significance of a certain stimulus impacts the switch costs when switching to versus switching away from the stimulus and the related task (Johnson, 2009; Paulitzki et al., 2008). For instance, in the study of Paulitzki et al. (2008), participants performed a cued alternating runs paradigm consisting of an emotionally aversive (spider: smooth *vs.* hairy) and a neutral (digit: odd *vs.* even) task. The results indicated lower switch costs when switching to the threat-related spider task than when switching to the neutral digit task. In addition, individual levels of fear correlated negatively with switch costs when participants switched from the digit task to the spider task, while when participants switched from the spider task to the digit task, the correlation between switch costs and the level of fear was positive. The authors interpreted their findings by assuming that the emotionally aversive stimuli facilitated task reconfiguration processes through faster activation of the task-set representation associated with the spider stimuli, and a slower deactivation of the task representation when the task was no longer relevant. Furthermore, the study of Paulitzki et al. (2008) illustrates that the effects vary with the expression of relevant personality traits. That is, individual differences in personality can have a moderating influence on the strength of the task-set activation and, therefore, on switch costs, if the content of the task is related to that particular personality trait.

Previous work on monetary incentives and personally relevant affective stimuli provided useful insights into the motivation-cognition interaction, but, it is unclear, if these effects would generalise to other domains and can be replicated with other kinds of stimuli and motivational manipulations. In the current study, we focused on the domain of eating and included images of food as task stimuli and a fasting manipulation in our experimental design. In the context of research on the motivational impact on task switching, the fasting manipulation is of particular interest, because hunger increases the positive valence of food (Stoeckel et al., 2007) and, therefore, can temporarily increase the motivational salience of food stimuli. In contrast to previous studies that investigated effects of affective-motivational stimuli with rather stable valence, our study focused on the motivational effects of food stimuli that can transiently change depending on hunger.

In fact, previous work has already demonstrated that hunger can have a significant moderating effect on attentional processing in tasks that contain food stimuli. For example, several studies that investigated attention allocation using the dot-probe task (Koster et al., 2006; Mogg et al., 2000) showed that hunger was associated with both faster engagement with food cues (Jonker et al., 2020; Mogg et al., 1998; Nijs et al., 2010) and more difficulties to disengage attention from these cues (Tapper et al., 2010). However, with regard to effects of fasting on the ability to switch between food and non-food stimuli, the pattern of findings is less consistent and not unequivocal. In one of the rare studies of this research vein, the study of Bolton et al. (2014) investigated the effects of fasting on cognitive flexibility in tasks involving food stimuli. In that study, participants were presented with sets of one to six images (food items vs. household objects), and categorised the number of presented images as odd or even or as low or high once in a sated state and once after 16 h fasting period. As a result, fasting led to larger switch costs, which, however, did not differ between food and neutral stimuli. Other studies, for example Pender et al. (2014), found increased switch costs in a task-switching situation requiring participants to categorise the number of non-food stimuli as odd or even or as low or high after 18 h fasting compared to control. In a study of Piech et al., (2009), participants identified by trial and error which object was the target in a stimulus set consisting of two faces and two buildings. After six correct responses, either the stimulus of another category became a target (rule change) or the stimulus set changed (set change); the authors varied hunger by a 5 h fasting manipulation and desire by presenting images of food items vs. flowers prior to completion of the task. As a result, hunger and desire affected independently the shifting performance, as indicated by higher error rates in shift trials to another category compared to the shift trials within the same category.

Although these studies indicate that both fasting and processing of food stimuli can impact task-switching performance, the results are not conclusive about a potential conjoined impact of fasting and food stimuli on shifting behaviour because in none of the tasks in the above-mentioned studies judgements about food stimuli were required. Food stimuli were either not relevant for the task rules at all (Bolton et al., 2014) or they were used as a tool to induce desire to eat prior to the completion of the task with non-food stimuli (Piech et al., 2009).

### The present research

The primary goal of the present study was to investigate the effects of food cues as task stimuli constituting one of the two tasks on the switching performance in situations requiring task switching in pre-determined (i.e., externally cued) order or in an order resulting from voluntary decisions. A further goal was to examine whether hunger as a motivational state variable would affect the effects of food stimuli on task-switching performance. To this end, participants performed two tasks: categorisation of food images as sweet or savoury (food task) and a neutral task, requiring the categorisation of digits as odd or even (digit task). Depending on the type of the task-switching condition, participants either performed the two tasks according to a pre-specified task order (AABBAA) in the alternating run procedure, or according to a self-determined order in the voluntary task switching procedure (see below, for the precise instruction). In addition, hunger was manipulated through 14 h fasting period in half of participants.

As a first hypothesis, we predicted the occurrence of differences in the switch costs associated with the food and the digit task. According to the findings of Paulitzki et al. (2008), we assumed that the presentation of food cues would result in facilitated control processes when switching to the food task. Based on their association with real food, food stimuli are supposed to act as a “magnet” and to trigger automatic approach tendencies towards them, which should further strengthen the activation of the food task set in working memory. In sum, these mechanisms are supposed to facilitate processes of reconfiguration of the food task set, which should result in lower switch costs when switching to the food task compared to the switch costs when switching to the digit task. On the other hand, in order to switch to the digit task, one needs to overcome the activation of the food task and activate the digit task. Given that the activation of the food task is supposed to be stronger than the activation of the digit task, switching to the digit task should require increased control, resulting in larger switch costs.

As a second hypothesis, we assumed that switch costs associated with each task would be modulated by hunger as a motivational state. Since motivational salience can vary depending on individuals' current needs, we expected that motivational effects of food stimuli would be magnified, if participants are hungry. Specifically, hunger should lead to even stronger activation of the task representation of the food task than the digit task, leading to lower switch costs when switching to the food task in fasted compared to sated participants. In contrast, when switching to the digit task, hunger should lead to even slower deactivation of the food task so that the switch costs associated with the digit task would be higher in fasted compared to sated participants.

In addition to examining the effects of fasting, we also investigated whether there is a relationship between the dynamics of switch costs and individual differences in participants' tendencies towards restrictive eating. For that purpose, participants conducted a brief self-report scale that addresses their eating patterns (Zickgraf & Ellis, 2018; for details see method and results section). Per analogy to the findings of Paulitzki et al. (2008), we asked whether individual differences in restrictive eating would be related to differences in switch costs for the different tasks. According to the activation assumption (Paulitzki et al., 2008), one should expect that higher individual restrictive eating tendencies would be associated with lower switch costs when switching to the food task and higher switch costs when switching to the digit task. Such result patterns would be consistent with the assumption that the biased processing of food stimulus information in individuals with higher compared to lower individual restrictive eating tendencies would be related to faster activation and slower deactivation of the food task set, leading to lower switch costs for the food task and higher switch costs for the digit task.

Additionally, we exploratory tested, whether the switch costs effects predicted in hypotheses 1 and 2 would depend on the specific task-switching paradigm. Since the predicted influences are assumed to be related to the mechanism of activation and deactivation of task sets in working memory, it seems reasonable to assume that these mechanisms do not systematically differ between externally cued and voluntary task switching. However, on the other side, fasting might lead to larger switch costs especially in cued task switching than in voluntary task switching because in the latter condition switching can be initiated in advance, which in turn, could facilitate the efficiency of processes involved in inhibiting the earlier task set and switching to the new task set and counteract the fasting-related decrease in flexibility (Bolton, et al., 2014; Pender et al., 2014; see also Arrington & Logan, 2004).

With regard to task selection in voluntary task switching, we refrained from drawing specific predictions about possible task preferences based on the nature of the tasks or the motivational states. Since in the present study we employed the classic instructions of Arrington and Logan (2005) asking participants to perform both tasks equally often and in a random order, it is unclear, if and how the manipulation of motivational salience of the tasks would modulate task selection.

## Method

### Participants

We conducted a power analysis with G*Power (Faul et al., 2007) to calculate the sample size for factors analyses of variance (ANOVAs) assuming a medium effect size and a standard two-tailed alpha value (*p* < 0.05) at a statistical power of 0.85. For that purpose, we determined the expected effect size based on the study of Bolton et al. (2014), who reported a medium effect size of a fasting manipulation on task shifting with fasting as a between subjects factor and trial as a within subjects factor. The remaining parameters included into the G*Power calculation were as follows: number of groups 2 (fasting, control) and number of measurements 2 (repetition, switching), correlation among repeated measurement 0.5, which resulted in a required sample size of 110 participants.

Because the current experiment was conducted as online experimentation (see below), which is characterized by increased dropout rates, we initially invited 125 participants of the Martin Luther University of Halle-Wittenberg for participation in the experiment. Exclusion criteria for the fasting condition were diabetes and acute and chronic diseases of the gastrointestinal tract. 10 participants were excluded from analysis, because their accuracy in either voluntary task switching or alternating run paradigm was below 85%. The data of further five participants were excluded, because their switch rate in voluntary task switching was extremely low (lower than 10%; see also Arrington et al., 2014). The final sample consisted of 110 participants, with 54 participants in the fasting condition and 56 participants in the control condition. Participants received a credit course for participation, or alternatively took part in the drawing of two vouchers of 30 Euros each.

### Fasting protocol

In the fasting condition, participants had to withdraw from food, coffee, and sugary drinks for 14 h prior to the experimental session. The fasting period took place overnight and lasted from 9:00 p.m. to 11:00 a.m. the following day. The experimental session in the fasting condition always started at 11 a.m. and lasted for approximately 1.25 h. Participants in the control condition did not receive any instruction concerning their meals. They could follow their natural eating rhythm. At the beginning of the experimental session, participants from the fasting condition were asked whether they adhered to fasting. To encourage participants to admit in the case that they had not followed the fasting protocol, they were informed that even if so, they still could participate in the study. All participants reported to have had adhered to fasting. Participants from both conditions were also asked how hungry they felt on the scale from 1 (*not hungry at all*) to 5 (*very hungry*). The hunger ratings of the control condition (*M* = 2.14, SD = 1.26) were significantly lower than the hunger ratings of the fasting condition (*M* = 3.56, SD = 0.95; *t*(109) = − 6.645, *p* < 0.001).

### Materials

Stimuli were digits from 1 to 9 (except for 5) and eight images of high palatable food (four savoury and four sweet dishes). We selected the food stimuli from the validated food image database for experimental research on eating and appetite (Blechert et al., 2019). The subset of savoury dishes included images no. 85, no. 145, no. 387, and no. 723. The sweet dishes subset contained images no. 6, no. 78, no. 139, and no. 140. The food stimuli were matched in valence, arousal, complexity, palatability and desire to eat that food if it was available.

Participants were instructed to categorise either food (sweet or savoury) or digits (odd or even) on each trial. Responses were made by pressing the 'd', 'f', 'j', and 'k' keys on the standard QWERTY keyboard. Both tasks were mapped to different fingers (index and middle) of the left and the right hand, respectively. The food task was mapped to the keys 'd' and 'k' and the digit task to the keys 'f' und 'j', category-to-hand mappings were counterbalanced across participants.

**Fig. 1:**
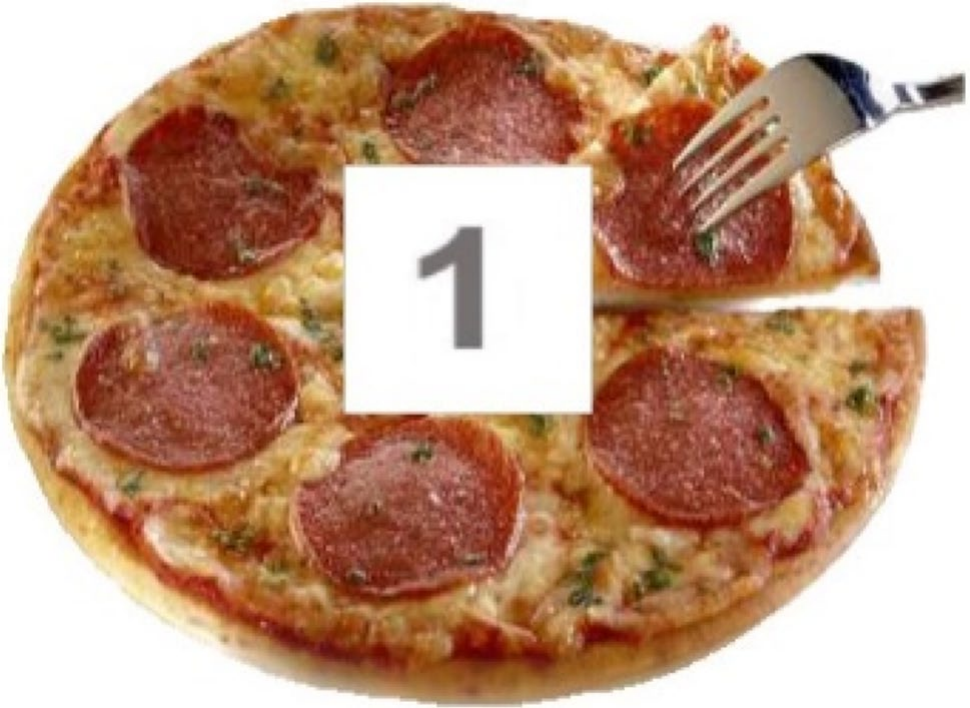
Example of compound food-digit stimulus

The stimuli were presented as compound stimuli. The choice of stimulus format was based on previous work (Johnson, 2009; Paulitzki et al., 2008) that used affective-motivational images at the background with digits or shapes placed in the centre of affective images. In our design, the digits were presented in grey on the background of white boxes overlaid on the image of a food stimulus. The box was displayed in the centre of the food image, as can be seen in Fig. 1. The size of the combined food-digit stimulus was scaled relative to the size of the participant's screen resolution in pixels (0.6 of the screen width, 0.5 of the screen height). Each trial started with the appearance of the fixation cross for 500 ms. Then, the combined food-digit stimulus was presented until a response was given or the maximum presentation time of 3000 ms elapsed. On each trial, participants received a feedback message “correct!” or “wrong!” for 500 ms, followed by the ITI of 500 ms. When maximum time elapsed without a response, the feedback message “to slow!” was shown. On every trial, the target digit and the food image appeared simultaneously. Each food image was combined with each digit (64 combinations in total). Throughout the experiment, each combination was presented eight times in random order.

### Restrictive eating

As a self-report scale, we used the Nine Item Avoidant/Restrictive Food Intake Disorder Screen (NIAS) (Zickgraf & Ellis, 2018). Avoidant/Restrictive Food Intake Disorder (ARFID) is an eating pathology that includes avoidance of certain foods based of their sensory characteristics, low appetite or lack of interest in eating, or fear of aversive consequences from eating (e.g. chocking, vomiting) (APA, 2013). The NIAS questionnaire consists of three subscales: Picky eating*,* Lack of appetite*,* and Fear of eating*,* each comprising three items*.* Picky eating is characterized by avoidance of particular colours, tastes, textures or smells and reluctance to try new foods (e.g., “I dislike most of the foods that other people eat.”). Lack of appetite refers to low homeostatic and hedonic appetite. Individuals with this symptom report to eat low amounts of food, and describe eating as a chore (e.g., “Even when I am eating a food I really like, it is hard for me to eat a large enough volume at meals.”). Fear of eating is related to earlier-experienced food-related trauma (e.g. chocking). As a result, certain foods or even groups of foods are avoided to prevent another negative experience (e.g., “I avoid or put off eating because I am afraid of GI discomfort, choking, or vomiting.”).

A six-point scale was used to rate how strongly participants agreed or disagreed with the statements, from “strongly disagree” to “strongly agree”. Subscale scores were obtained by summing up three items from each scale, a total score was calculated by summing up all items.

### Procedure

Participants completed the study online. The experiment was implemented using PsychoPy (Peirce & MacAskill, 2018) software for psychological experiments and was made available for participants by hosting on the server (http://www.pavlovia.org).

Self-paced instructions to both variants of task switching were presented on the screen. In the voluntary task switching, participants were instructed to freely select which task to perform on each trial. They were told to try to perform both tasks equally often in random order. According to the procedure of Arrington and Logan (2004, 2005), we further clarified that participants should avoid any rules or recurring sequences when selecting a task. In the alternating runs switching, participants were required to switch between the tasks every second trial. The current task was, thus, indicated by its (temporal) position of the current trial within the predictable 4-trials run (AABB). Each procedure included one practice block of 64 trials and 4 test blocks of 64 trials each. After each block, a short break was administered to participants. Participants first completed voluntary task switching followed by alternating runs switching. After completing both task-switching paradigms, participants filled out the NIAS questionnaire.

### Data processing

In both task-switching conditions, we categorised trials as task repetitions and task switches, depending on the task performed in trial n and trial n-1. In voluntary task switching, the responses were assigned to the tasks based on the fingers participants used to perform the task. A trial was coded as an error if the given response was not correct for the task as determined by the fingers. In both conditions, the first trial of each block, error trials and trials following errors were eliminated in all reaction time (RT) analyses. This led to the removal of 12.27% of the data in voluntary task switching. The data loss in alternating runs switching was 14.08%. For error analyses, only the first trial of each block was excluded.

For the subsequent analyses, we calculated switch costs separately for the food and for the digit task. The food-task switch costs were calculated by subtracting median RTs in food task repetition trials from median RTs in switch trials when switching to the food task. The digit-task switch costs were obtained by subtracting median RTs in digit task repetitions from median RTs in switch trials when switching to the digit task.

## Results

We first run an overall omnibus ANOVA on the performance of participants in the alternating runs paradigm and the voluntary task switching paradigm together to get a general overview about the results on both paradigms. Subsequently, we conducted separate analyses for the alternating runs task switching and the voluntary task switching paradigm, which allowed us to investigate the hypotheses about the impact of motivational-affective task stimuli and about the fasting condition on the switching processes in more detail for the two separate switching situations.

### Overall analysis

We conducted a 2 (task: food task vs. digit task) × 2 (trial type: repetition vs. switch) × 2 (paradigm: alternating runs switching vs. voluntary task switching) × 2 (condition: fasting vs. control) mixed factors analysis of variance (ANOVA) on pooled data of participants in the two paradigms. We did so in order to test for general effects of motivational salience of the tasks and fasting condition on participants’ performance.

For RTs, the analyses showed a main effect of paradigm, *F*(1,108) = 72.920, *p* < 0.001, *η*_p_^2^ = 0.019, reflecting significantly slower RTs in voluntary task switching (*M* = 660 ms) compared to alternating runs task switching (*M* = 612 ms), *t*(109) = 8.705, *p* < 0.001. The analyses further revealed a significant paradigm × task interaction, *F*(1,108) = 11.979, *p* < 0.001, *η*_p_^2^ = 0.100, indicating that the RT difference between the paradigms was slightly greater for the digit task (*M* = 64 ms) than for the food task (*M* = 38 ms; *t*(109) = 4.116, *p* < 0.001). Furthermore, the RT difference between the tasks was greater in voluntary task switching (*M* = 122 ms) than in alternating runs task switching (*M* = 96 ms), *t*(109) = 4.116, *p* < 0.001. Despite overall higher RTs in voluntary task switching, the switch costs were significantly larger in alternating runs switching (*M* = 108 ms) compared to voluntary task switching (*M* = 62 ms), *t*(109) = 5.538, *p* < 0.001, as reflected by the significant paradigm × trial interaction, *F*(1,108) = 37.824, *p* < 0.001, *η*_p_^2^ = 0.259. This interaction additionally differed across the levels of the factor condition, i.e. paradigm × trial × condition, *F*(1,108) = 6.152, *p* < 0.05, *η*_p_^2^ = 0.054. This reflects the observation that the fasting-related increase of switch costs was especially large in the alternating runs paradigm (fasting: *M* = 121 ms, control: *M* = 89 ms; *t*(108) = 2.081, *p* < 0.05) as compared to the voluntary task switching paradigm (fasting: *M* = 53 ms, control: *M* = 70 ms; *t*(108) = − 1.263, *p* > 0.2), which will be specified in separate analyses for the alternating runs task switching and the voluntary task switching paradigms below. The effects of food stimuli and hunger did not differ between the paradigms, as neither the three-way task × trial × paradigm interaction, *F*(1,108) = 2.059, *p* = 0.154, *η*_p_^2^ = 0.019, nor the four-way task × trial × condition × paradigm interaction, *F*(1,108) = 0.619, *p* = 0.433, *η*_p_^2^ = 0.006, proved significant.

Corresponding analyses of error rates yielded a main effect of paradigm, *F*(1,108) = 17.387, *p* < 0.001, *η*_p_^2^ = 0.139, which was further qualified by significant paradigm × task, *F*(1,108) = 6.345, *p* < 0.05, *η*_p_^2^ = 0.055, and paradigm × trial, *F*(1,108) = 21.739, *p* < 0.001, *η*_p_^2^ = 0.168, interactions. The difference in error rates between food task and digit task was greater in voluntary task switching (*M*_food_task_ = 2.8%; *M*_digit_task_ = 8.0%; *t*(109) = − 11.483, *p* < 0.001) compared to alternating runs task switching (*M*_food_task_ = 4.6%; *M*_digit_task_ = 8.9; *t*(109) = − 11.084, *p* < 0.001). Further, error rates differed significantly between the two paradigms only for the food task (*M*_food_task_vts_ = 2.8%; *M*_food_task_ars_ = 4.6%; *t*(109) = − 5.994, *p* < 0.001). For the digit task, the difference was only marginally significant (*M*_digit_task_vts_ = 8.0%; *M*_digit_task_ars_ = 8.9%; *t*(109) = − 1.975, *p* = 0.05). As suggested by the paradigm × trial interaction, *F*(1,108) = 21.739, *p* < 0.001, *η*_p_^2^ = 0.168, participants also made more errors in switch trials in alternating runs task switching (8.6%) than in voluntary task switching (6.3%), *t*(109) =  − 5.471, *p* < 0.001. In contrast, error rates did not differ between the paradigms in repetition trials (*M*_rep_vts_ = 5.1%; *M*_rep_ars_ = 5.0%; *t* < 1, n.s.). The switch costs were significantly larger in alternating runs task switching (3.6%) than in voluntary task switching (1.3%), *t*(109) = 5.448, *p* < 0.001. The difference in the switch costs was not further modulated by task or condition, as indicated by non-significant three-way task × trial × paradigm interaction, *F*(1,108) = 1.973, *p* = 0.163, *η*_p_^2^ = 0.018 and four-way task × trial × condition × paradigm interaction, *F*(1,108) = 0.794, *p* = 0.375, *η*_p_^2^ = 0.007. Together, these results suggest that effects of motivational salience on switch costs are valid for both, the alternating runs switching and the voluntary task-switching paradigm.

Next, we analysed the effects of motivational task salience and fasting condition separately for the two switching paradigms.

### Alternating runs switching

#### Hypothesis 1

To analyse in more detail, the switch costs in the alternating runs paradigm and its modulation by the motivational salience of the tasks, we subjected the alternating runs RTs of participants to a separate 2 (task: food task vs. digit task) × 2 (trial type: repetition vs. switch) × 2 (condition: fasting vs. control) mixed factors ANOVA. The same analysis was conducted with error rates. The first hypothesis predicted lower switch costs when switching to the food task compared to when switching to the digit task. This effect should be reflected in a significant task × trial interaction in the analyses of RTs and error rates.

### RT analysis

Indeed, RTs in the alternating runs condition varied as a function of task and trial type, as indicated by main effects of task, *F*(1,108) = 324.673, *p* < 0.001, *η*_p_^2^ = 0.750, trial type, *F*(1,108) = 243.499, *p* < 0.001, *η*_p_^2^ = 0.693 and their interaction, *F*(1,108) = 93.293, *p* < 0.001, *η*_p_^2^ = 0.463. Post-hoc comparisons revealed smaller RTs for the food task repetitions (*M* = 543 ms) than for the food task switches (*M* = 610 ms), *t*(109) = 15.384, *p* < 0.001. The digit task repetitions (*M* = 606 ms) were also faster than digit task switches (*M* = 773 ms), *t*(109) = 13.339, *p* < 0.001. As predicted, the switch costs for the food task (*M* = 67 ms) were significantly smaller than the switch costs for the digit task (*M* = 168 ms), *t*(109) =  − 9.409, *p* < 0.001, indicating that participants needed less time when switching to the food task than when switching to the digit task, which is consistent with the first hypothesis. The switch costs difference between the two task conditions was *M* = 101 ms.

### Error rates

The analyses of error rates showed a similar pattern of results, as indicated by main effects of task, *F*(1,108) = 119.947,* p* < 0.001, *η*_p_^2^ = 0.526, trial type, *F*(1,108) = 104.379, *p* < 0.001, *η*_p_^2^ = 0.491, as well as their interaction, *F*(1,108) = 53.334, *p* < 0.001, *η*_p_^2^ = 0.331. Post-hoc comparisons indicated that participants made fewer errors in food task repetitions (*M* = 4.1%) than in food task switches (*M* = 5.1%), *t*(109) = 2.689, *p* < 0.01. Similarly, participants made fewer errors in digit task repetitions (*M* = 5.8%) than in digit task switches (*M* = 11.9%), *t*(109) =  − 10.376, *p* < 0.001. The switch costs difference between the food task (*M* = 1.0%) and the digit task (*M* = 6.1%) was significant *t*(109) =  − 7.214, *p* < 0.001, suggesting that participants made fewer errors when switching to the food task than when switching to the digit task. Thus, differences in motivational salience of the tasks led to lower switch costs when switching to the food task and to higher switch costs when switching to the digit task, supporting the first hypothesis.

#### Hypothesis 2

To test the second hypothesis, we analysed the above-mentioned task × trial interaction in the fasting and in the control condition of the alternating runs paradigm with a 2 (task: food task vs. digit task) × 2 (trial type: repetition vs. switch) × 2 (condition: fasting vs. control) mixed factors ANOVA. Of primary interest was the three-way task × trial × condition interaction for RTs and error rates. According to hypothesis 2, the task × trial interaction effect should be larger in the fasting than in the control condition.

### RT analysis

**Fig. 2:**
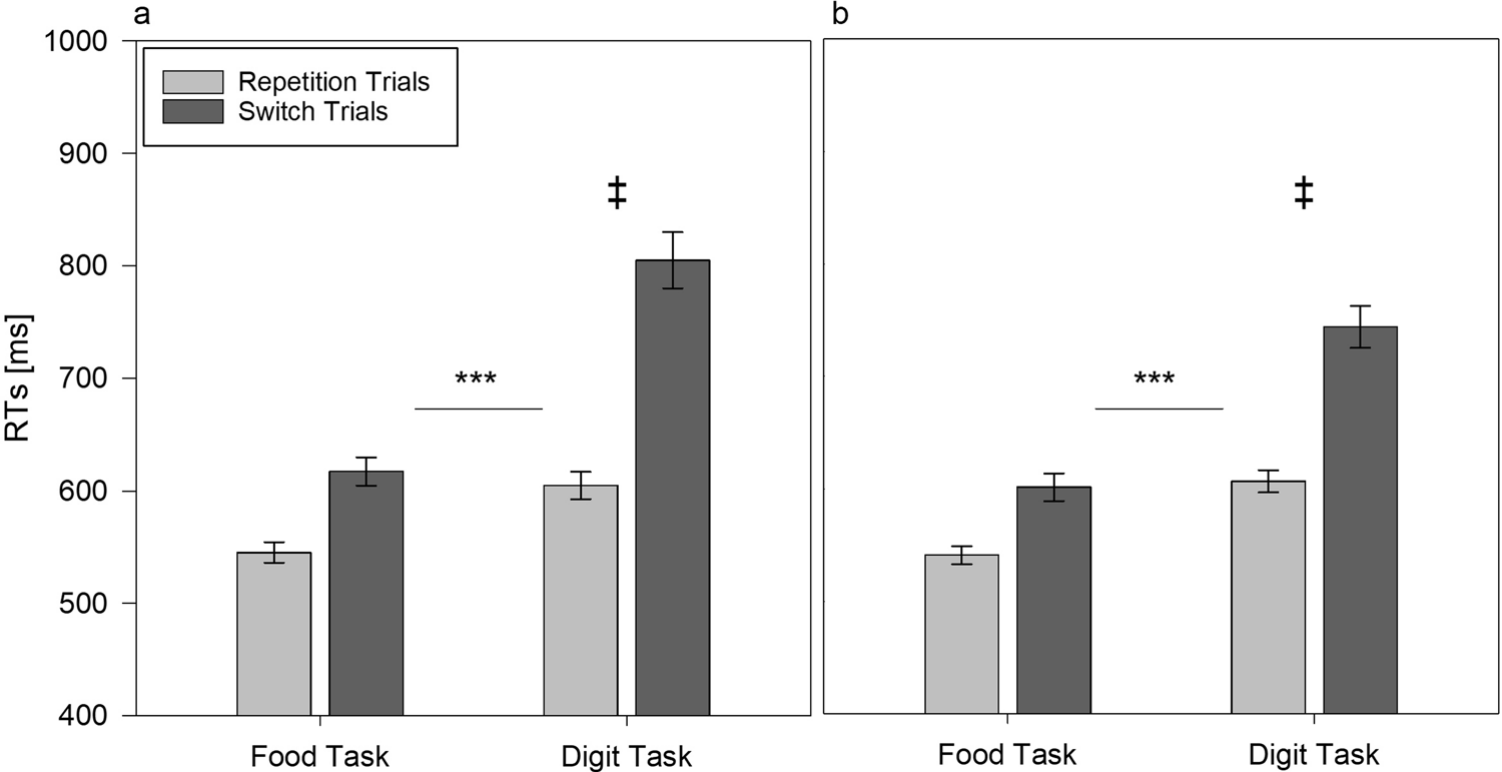
Response times (medians) averaged across participants depending on task and trial type in alternating runs task switching in fasting (**a**) and in control condition (**b**). Error bars represent standard errors of mean. ***Difference in switch costs between the food task and the digit task, *p* < 0.001. ^**‡**^Difference in digit task switch costs between fasting and control condition, *p* < 0.05

Indeed, the analysis of RTs showed a significant three-way task × trial × condition interaction *F*(1,108) = 5.864, *p* = 0.017, *η*_p_^2^ = 0.051. As can be seen in Fig. 2a, b, the effect of fasting on the switch costs was stronger for the digit task compared to the food task. This is supported by the results of the separate comparisons of the RTs in the digit task and the food task. For the digit task, these comparisons showed no differences between conditions for the repetition trials (fasting: *M* = 605 ms, control: *M* = 607 ms, *t*(109) = 0.122, *p* = 0.903), while the digit task switch RTs were larger for the fasting condition (*M* = 805 ms) compared to the control condition (*M* = 744 ms), *t*(109) = 1.962, *p* = 0.05. Therefore, for the digit task, the switch costs were larger in the fasting condition (*M* = 128 ms) compared to the control condition (*M* = 77 ms), *t*(109) = 2.421, *p* = 0.017. For the food task, we found no differences between fasting and control condition for food task repetitions (fasting: *M* = 545 ms, control: *M* = 541 ms, *t*(109) = 0.326, *p* = 0.745) and food task switches (fasting: *M* = 617 ms, control: *M* = 602 ms, *t*(109) =  − 0.894, *p* = 0.373).

In sum, fasted participants have more difficulty to discard the food task in order to switch to the digit task than sated control participants.

### Error rates

**Fig. 3:**
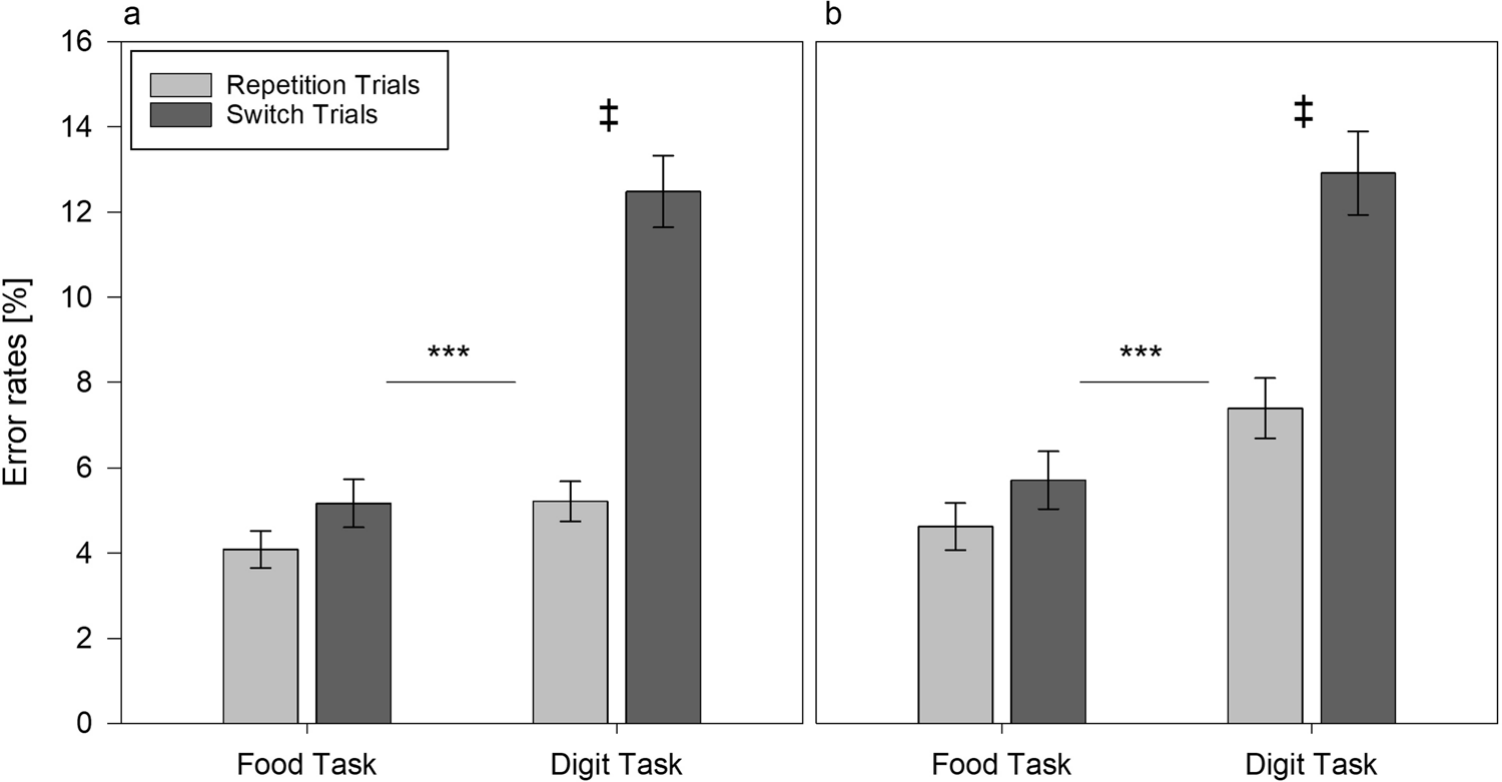
Average error rates (%) as a function of task and trial type in alternating runs task switching in fasting (**a**) and in control condition (**b**). Error bars represent standard errors of mean. ***Difference in switch costs between the food task and the digit task, *p* < 0.001. ^**‡**^Difference in digit task switch costs between fasting and control condition, *p* < 0.05

We found a non-significant task × trial × condition interaction for the error rates *F*(1,108) = 2.818, *p* = 0.096, *η*_p_^2^ = 0.025. None of the direct post-hoc comparisons between the error rates in repetition and switch trials between conditions proved significant (all *p*'s > 0.328). However, post hoc comparisons revealed that the patterns of switch costs (i.e., the difference between error rates in switch and repetition trials), were similar for the error rates as for the RT results; that is, we found a larger error rate difference in switch costs between the fasting (*M* = 7.3%) and the control condition (*M* = 4.8%) for the digit task, *t*(108) =  − 2.167, *p* = 0.032, while we did not find an error rate difference between fasting (*M* = 1.13%) and control condition (*M* = 0.95%) for the food task, *t*(108) =  − 0.222, *p* = 0.825 (Fig. 3a, b). Thus, in sum, the results of the RT and the error rate analyses provided partial support for the second hypothesis, indicating larger digit task switch costs in the fasting compared to the control condition.

### Voluntary task switching

#### Hypothesis 1

To analyse in more detail, the switch costs in situations requiring voluntary task switching and its modulation by the motivational salience of the tasks, we subjected RTs and error rates in the voluntary task switching to a separate 2 (task: food task vs. digit task) × 2 (trial type: repetition vs. switch) × 2 (condition: fasting vs. control) mixed factors ANOVA.

### RT analysis

Similar to the alternating runs switching results, the RT analyses yielded main effects of task, *F*(1,108) = 346.748, *p* < 0.001, *η*_p_^2^ = 0.763, trial type, *F*(1,108) = 111.386, *p* < 0.001, *η*_p_^2^ = 0.508, and a significant task × trial type interaction, *F*(1,108) = 67.697, *p* < 0.001, *η*_p_^2^ = 0.385. Post hoc comparisons revealed smaller RTs for the food task repetitions (*M* = 602 ms) than for the food task switches (*M* = 630 ms), *t*(109) =  − 5.820, *p* < 0.001. The digit task repetitions (*M* = 698 ms) were also faster than digit task switches (*M* = 810 ms), *t*(109) =  − 10.272, *p* < 0.001. Switch costs when switching to the food task (*M* = 28 ms) were significantly smaller than switch costs when switching to the digit task (*M* = 112 ms), *t*(109) =  − 8.143, *p* < 0.001, which is consistent with the first hypothesis suggesting lower switch costs when participants switch to the food task compared to the digit task. The switch costs difference was *M* = 84 ms.

### Error rates

The analyses of error rates showed a similar pattern of results, as indicated by main effects of task, *F*(1,108) = 123.715, *p* < 0.001, *η*_p_^2^ = 0.534, trial type, *F*(1,108) = 18.964, *p* < 0.001, *η*_p_^2^ = 0.149, as well as their interaction, *F*(1,108) = 26.442, *p* < 0.001, *η*_p_^2^ = 0.197. Error rates did not differ between food task repetitions (*M* = 3%) and food task switches (*M* = 2.6%), *t*(109) = 1.082, *p* = 0.223, while error rates were significantly lower in digit task repetitions (*M* = 6.6%) than in digit task switches (*M* = 10%), *t*(109) =  − 5.271, *p* < 0.001. The switch costs difference between the food task (M = − 0.4%) and the digit task (*M* = 3.4%) was significant, *t*(109) =  − 5.153, *p* < 0.001. These data indicate the observation of a modulatory effect of motivational salience on the switch costs also for the voluntary task switching paradigm.

#### Hypothesis 2

**Fig. 4:**
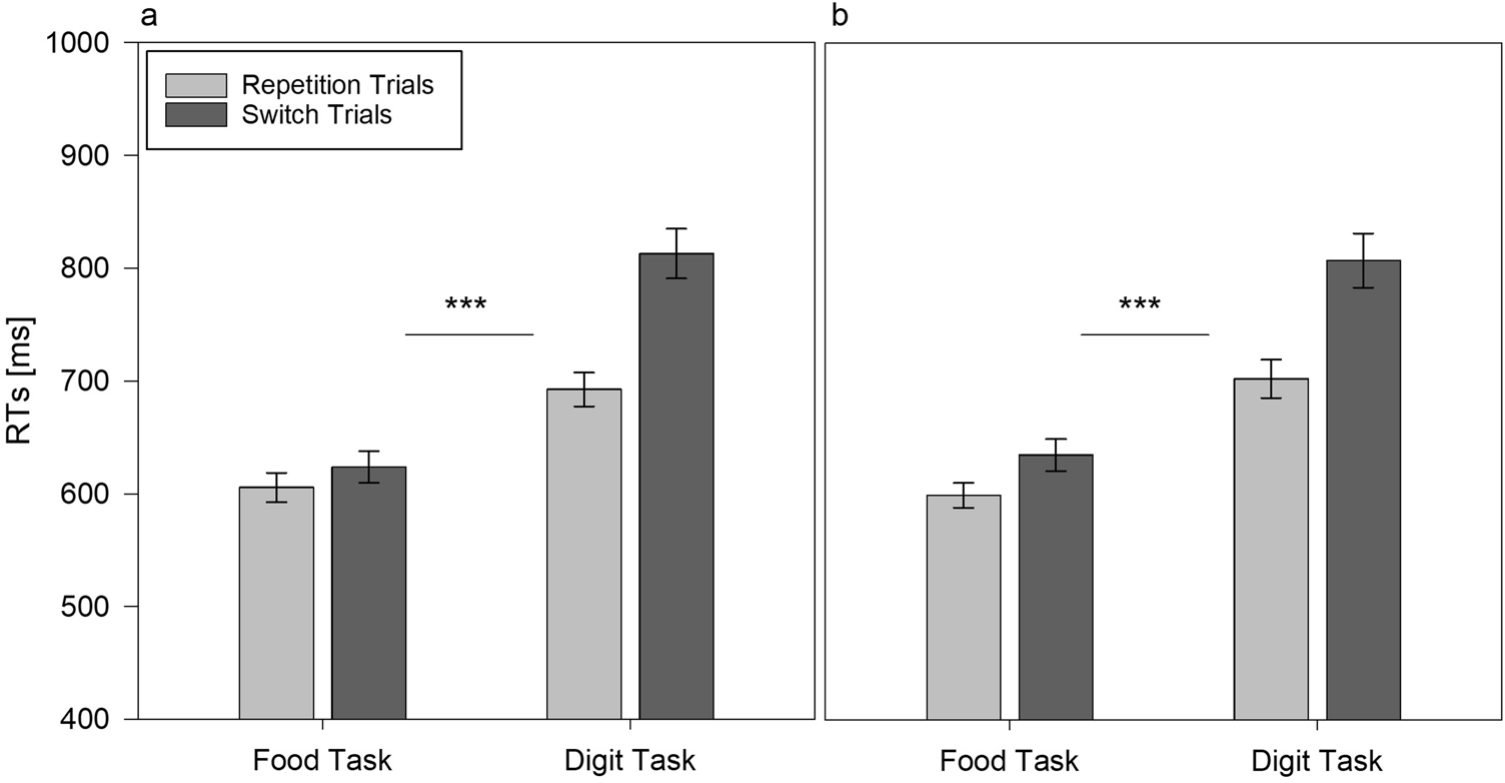
Response times (medians) averaged across participants depending on task and trial type in voluntary task switching in fasting (**a**) and in control condition (**b**). Error bars represent standard errors of mean. ***Difference in switch costs between the food task and the digit task, *p* < 0.001

**Fig. 5:**
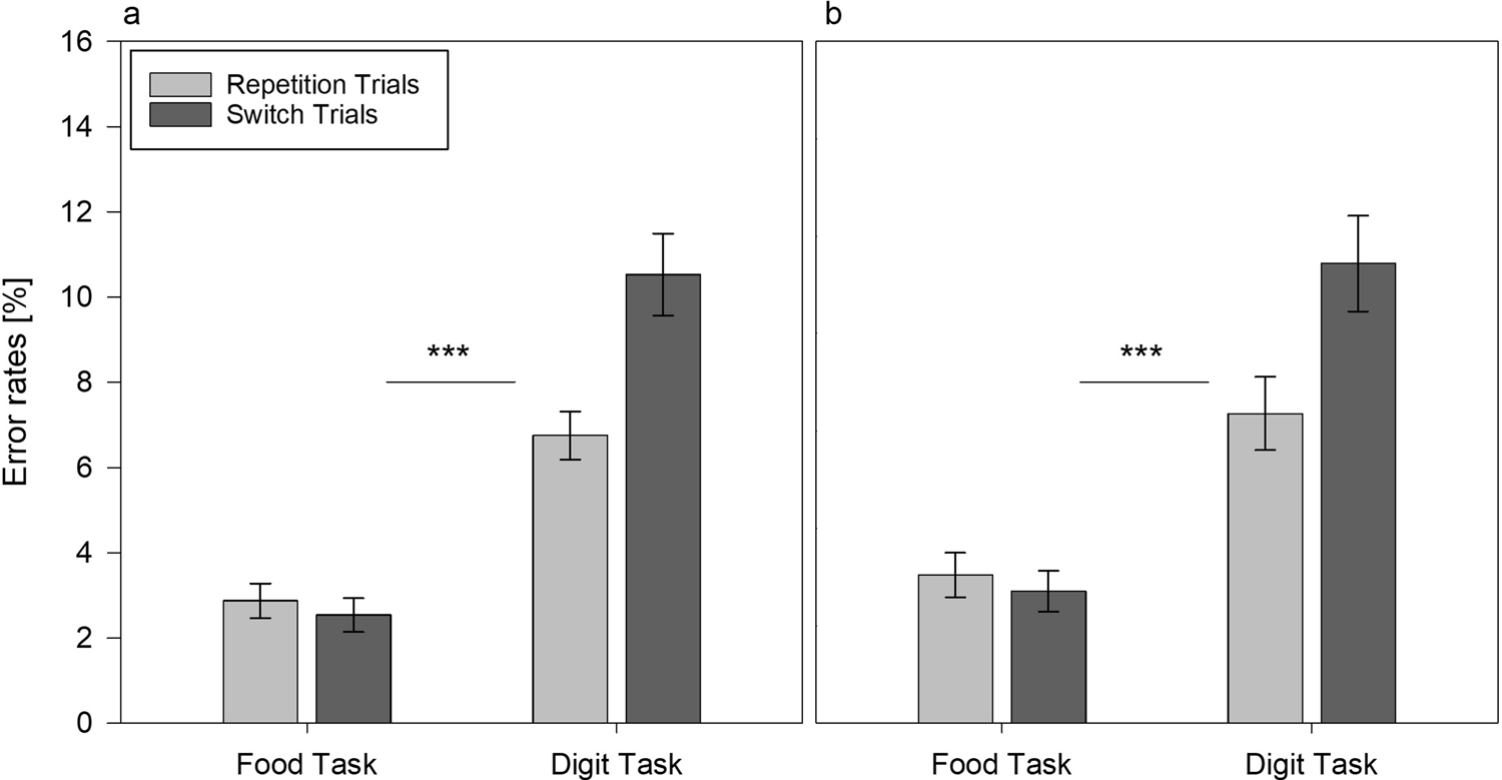
Average error rates (%) as a function of task and trial type in voluntary task switching in fasting (**a**) and in control condition (**b**). Error bars represent standard errors of mean. ***Difference in switch costs between the food task and the digit task, *p* < 0.001

To test the second hypothesis for the voluntary task switching performance, we subjected the corresponding RTs and error rates to a 2 (task: food task vs. digit task) × 2 (trial type: repetition vs. switch) × 2 (condition: fasting vs. control) mixed factors ANOVA. The analysis of RTs showed that the three-way task × trial × condition interaction was not significant, *F*(1,108) = 2.528, *p* = 0.115, *η*_p_^2^ = 0.023 (Fig. 4a, b), which reflects the fact that the switch costs did not differ between the fasting condition (*M* = 53 ms) and control condition (*M* = 70 ms), although both proved significant for the two conditions, fasting, *t*(53) =  − 7.358, *p* < 0.001, and control, *t*(55) =  − 6.363, *p* < 0.001, respectively. For error rates, we also did not find a significant interaction, *F* < 1, (Fig. 5a, b).

### Task choice in voluntary task switching

In general, participants chose to repeat tasks (*M* = 0.591) more often than to switch tasks (*M* = 0.409), *t*(108) = 33.970, *p* < 0.001, which replicates the repetition bias, a robust finding in the voluntary task switching literature (Arrington et al., 2014). To explore, whether motivational salience was associated with differences in the probabilities for selecting tasks, we calculated the probabilities for selecting each task across task repetitions and task switches. The comparison of these probabilities showed that overall participants complied with the instruction to select both tasks equally often. The mean probability for choosing the food task was *p*_choice_ = 0.493, which was not significantly different from *p*_choice_ = 0.5, *t*(109) =  − 1.389, *p* = 0.168. The mean probability for choosing the digit task was *p*_choice_ = 0.507. We also tested, whether task choice probabilities differed between fasting conditions. These analyses showed no significant task choice bias in either of the conditions. The probability of trials on which the food task was chosen in the fasting condition amounted to *p*_fasting_ = 0.486 and to *p*_control_ = 0.499, which did not differ significantly, *t*(108) = 1.242, *p* = 0.217; choice probability for neither condition was significantly different from chance, fasting: *t*(53) =  − 1.901, *p* = 0.063; control: *t*(55) =  − 0.121, *p* = 0.904. We further examined, if there were differences in task choice probabilities, when the probabilities for each task were further subdivided in task repetitions and task switches. For that purpose, we ran ANOVAs with the within-subjects factor task (food vs. digit) and the between-subjects factor condition (fasting vs. control) separately for the probabilities of task repetitions and switches. For the task repetitions, participants showed slightly higher probabilities for selecting the food task (*p*_choice_ = 0.305) compared to the digit task (*p*_choice_ = 0.297) although this effect was not significant, *F*(1,108) = 3.131, *p* = 0.08, *η*_p_^2^ = 0.028. All other comparisons showed no systematic choice biases depending on task, condition, and their interactions, largest *F*(1,108) < 1, largest *p* > 0.35. In sum, we did not detect any impact of the motivational nature of the tasks and the fasting condition on task selection in voluntary task switching.

### Correlational analyses

Finally, we analysed whether individual differences in ARFID are related to the switch costs for the food task and the digit task and conducted correlational analyses for each type of switch costs and the total score on the NIAS scale. For that analysis, we collapsed together the switch costs for the RT data and for the error rates across both paradigms and represent them as an average of switch costs in voluntary task switching and alternating runs switching. This approach is justified because voluntary task switching and alternating runs switching did not differ in terms of the relevant task × trial interactions and the task × trial × condition interactions, which did not interact with the factor paradigm in the overall analysis, (see overall analysis before, both *F*'s < 1.97, both *p*'s > 0.15).

**Fig. 6:**
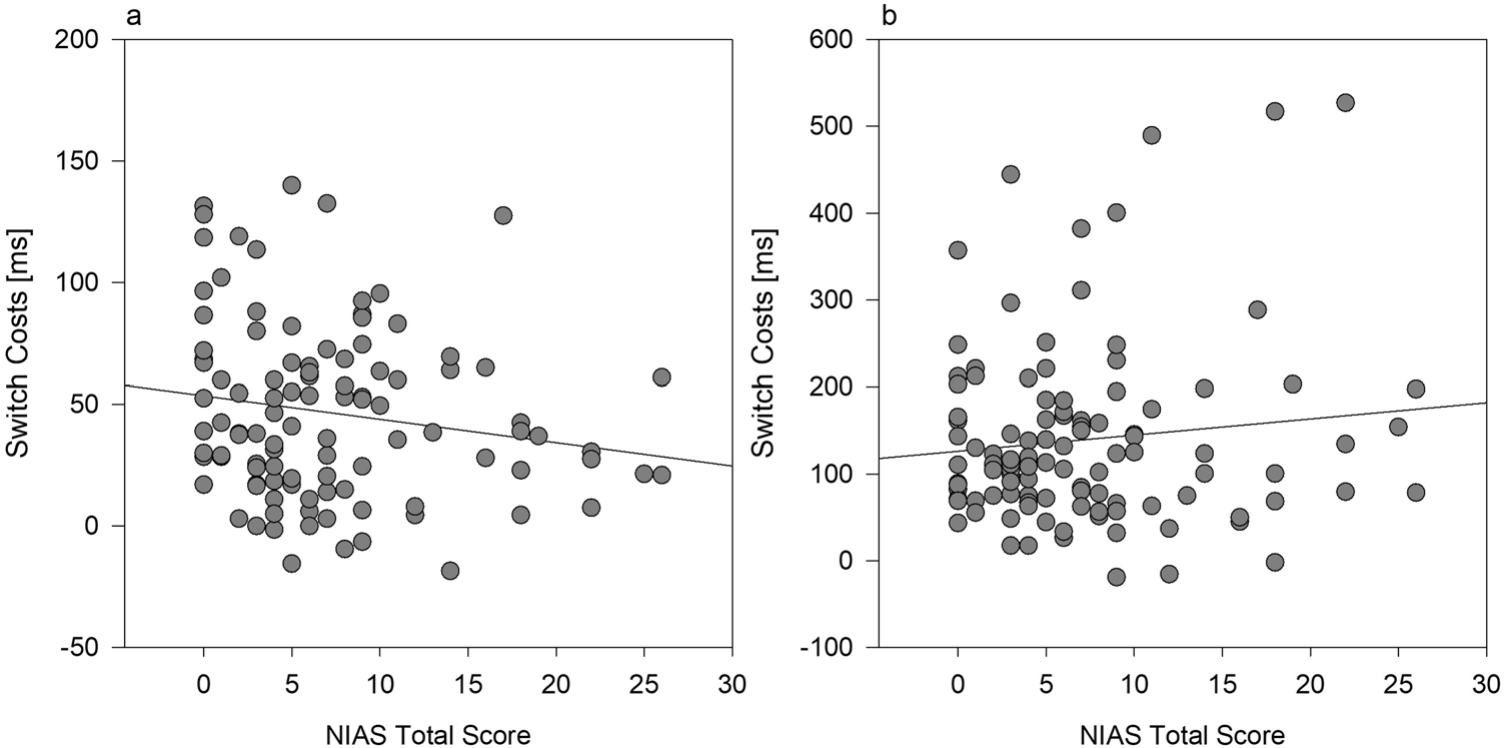
Correlations between switch costs for the food task (**a**) and for the digit task (**b**) and NIAS total scores. Higher values indicate higher levels of restrictive eating

If the biased processing of food stimulus information in individuals with higher compared to lower NIAS scores would be related to faster activation and slower deactivation of the food task set, then this should lead to lower switch costs for the food task and higher switch costs for the digit task with increasing values of NIAS. For the case of RTs, the total NIAS scores correlated negatively with the amount of switch costs for the food task, indicating that participants with higher levels of avoidant/restrictive eating performed faster when switching to the food task, *r* = − 0.168, *p* = 0.082 (see Fig. 6a). For the digit task, RT-related switch costs tended to be larger with increasing values of NIAS, which is consistent with the prediction that participants have more difficulties to switch to the digit task with increasing degree of ARFID, although the correlation was not significant, *r* = 0.111,* p* = 0.25 (see Fig. 6b). The analogous analyses for error rates revealed no significant correlations for both task types (both *r*'s < 0.036, both *p*'s ˃ 0.357).

## Discussion

The impact of motivation on the ability to flexibly switch between the tasks has recently become a subject of increased scientific inquiry. Using affective-motivational stimuli, some researchers found evidence that the individual relevance of task stimuli influences cognitive control in task switching (Paulitzki et al., 2008). The present study investigated the motivational-affective influence on cognitive control during switching between a task with motivationally salient food stimuli and a neutral digit categorisation task, while relevance of the food stimuli was manipulated by hunger in one of the two groups of participants.

### Food task stimuli and task switching

In the first hypothesis, we predicted that switch costs when switching to a motivationally salient food task would be smaller than switch costs when switching to a neutral digit task. The pattern of results confirmed that hypothesis. As expected, participants switched faster to the motivationally salient food task, whereas the switching to the neutral digit task was slower. The observed performance benefit when switching to the food task is consistent with the assumption that motivation leads to faster activation of the food task set in working memory, facilitating task-set reconfiguration and resulting in lower switch costs. In order to switch to a task, participants need to select and to coordinate the appropriate processes, including stimulus categorisation, decision-making, mapping the stimulus' attribute to a reaction set category, and execution of the motor response (Rogers & Monsell, 1995). The current findings are consistent with the assumption that motivational salience contributes to the strength of the representation of the task, which in turn leads to an acceleration of the processes that take place during task-set reconfiguration. This is an important finding, because, to our knowledge, there have been quite a few studies that examined cognitive control in task switching using non-monetary motivation manipulations (e.g., Han et al., 2021). Compared with earlier studies on monetary reward, our results suggest that enhancing effects of food stimuli are comparable to performance effects of monetary incentives. Nevertheless, it is worth directly comparing effects of monetary rewards and stimuli linked to primary reinforcers under similar conditions. With regard to affective-motivational stimuli, our findings replicate the results of Paulitzki et al. (2008) who reported lower switch costs when switching to the threat-related spider-task compared to a neutral digit task, and the broader literature on visual attention demonstrating faster attentional engagement with motivationally salient food stimuli (see Werthmann et al., 2015 for a review).

It has to be noted though that the observed affective-motivational effects of food stimuli on the switch costs might be confounded with the effects of certain stimulus characteristics, which differed between food and digit stimuli. In more detail, food stimuli were larger and visually more complex than digit stimuli, which might have caused improved visual-attentional processing of these stimuli. However, an account assuming a pure visual-attentional effect on the RT performance would require an equal reduction of the RTs for the repetition and the switch trials in the food compared to the digit task. This was not the case in the current study. Here, the RT differences between the food and the digit tasks were larger for the task switch than for task repetition trials (both *p*’s < 0.001), which speaks against an explanation assuming that switch costs differences between tasks stem purely from the visual-attentional differences between task stimuli (see also Paulitzki et al., 2008). Besides that, we ran additional pilot experiments (unpublished data) that also required categorisation decisions for food stimuli and on the number of stimuli but in which the stimulus characteristics were balanced in terms of size and complexity; for example, consider a task with food stimuli presented in varying number (i.e. 1, 2, 3 or more food stimuli) and requiring food identity and stimulus number decisions. In these experiments, we also observed smaller switch costs for the affective-motivational task compared to the neutral number task, which shows that the differences in the switch costs can emerge even when controlling for perceptual characteristics of the stimuli for both tasks.

### Fasting

According to the second hypothesis, switch costs associated with each task should be modulated by hunger as a motivational state and the motivational effects of food stimuli should be magnified, if participants are hungry. Therefore, we expected the switch costs to be smaller when switching to the food task and the switch costs when switching to the digit task to be larger in the group of fasted participants compared to the control group. These predictions were partially supported. Consistent with the prediction, the costs when switching to the digit task were larger in the fasting condition compared to the control condition, which is consistent with the assumption that the activation of the food task set was stronger in the fasting condition than in the control condition. In that case, the deactivation of the food task set requires more time and effort when switching to the digit task, resulting in larger switch costs for that task after fasting.

Note, that this effect cannot be explained by generally slower RTs for the digit task in fasting compared to control condition, because the RT difference in repetition trials between fasting and control condition amounted to negligible 2 ms, whereas the RT difference in switch trials amounted to 61 ms. Although the latter RT difference only approached statistical significance (i.e., *p* = 0.05), a direct comparison of the switch costs showed significantly larger digit task switch costs under the fasting compared to the control condition. This is suggestive for an influence of fasting on switching to the digit task, although that result requires further replication, because it was observed only for the alternating runs condition.

We did not find significantly smaller switch costs for the food task in the fasting compared to the control condition. In both eating conditions, the switch costs for the food task were lower than the switch costs for the digit task and not modulated by fasting. One possible explanation for a lacking effect of fasting on the lower switch costs of the food compared to the digit task are floor effects: the high motivational salience of the food task and the respective food stimuli benefit performance in this task irrespective of the fasting condition. Because participants switched quickly to the food task, there is no room left for the switch costs in the fasting condition to improve. It is also conceivable that effects of fasting were masked by desire to eat the depicted food if it was available. The desire to eat or appetite emerges as a response to the presence of food even in a sated state of participants (e.g. Feig et al., 2018) and varies with individual food preferences and pleasure received from consuming high-caloric food. Since we used highly palatable food images as stimuli for the food task and did not control for individual desire to eat, we cannot rule out that fasting and control group did not differ in terms of desire to eat. Thus, food stimuli may have produced similar feelings of desire-to-eat, 'wanting' or 'liking' the food in both conditions (Piech et al., 2009), but only hungry participants had more difficulty to discard the food task representation in order to switch to the digit task. Comparable patterns of results were shown in several studies on attentional bias showing that hunger was associated with slower disengagement rather than faster engagement with food cues (Castellanos et al., 2009; Tapper et al., 2010), whereas effects of food cues on attentional engagement were found even in the absence of hunger (Pool et al., 2016).

We cannot rule out that a stronger fasting manipulation would have evoked improved switching to the food task as compared to the neutral digit task. Although participants from the fasting condition reported to be hungrier than the participants from the control condition, the difference in the levels of hunger might have been not strong enough for the predicted effects of fasting to fully unfold. Importantly, in literature, the findings on the effects of fasting duration on cognitive performance are inconsistent. Some studies have demonstrated fasting effects already after 10–12 h fasting (Ginieis et al., 2018; Macpherson et al., 2015), whereas others utilized much longer fasting protocols ranging up to 48 h (Solianik, et al., 2020, see Benau et al., 2021 for review). Regarding cognitive flexibility, results showed that hunger had no effect following 5 h fasting (Piech et al., 2009), but lead to performance impairment after 16 h (Bolton et al., 2014) and after 18 h (Pender et al., 2014). Following the latter studies, future research might consider prolonging the fasting duration in order to examine if the lack of differences between conditions can be explained by too short fasting duration.

### Fasting and task content in alternating runs and voluntary task switching

The comparison of the conjoint influence of fasting and motivational-affective task content on the alternating runs switching and the voluntary task switching yielded no evidence for differences between the paradigms, as indicated by the non-significant task × trial × paradigm × condition interaction. These findings suggest that the food-related and fasting-related motivational impact on cognitive control in task switching does not decisively differ between the way how switching is operated, i.e. either by external or internal cues. Fasting only increased switch costs in the alternating runs to larger extent than in the voluntary task switching paradigm, which is consistent with the assumption that fasting impairs cognitive flexibility by lowering the efficiency to de-activate the old task set and to re-configure new task sets on cue presentation in working memory (Bolton et al., 2014; Pender et al., 2014). We further detected a significant interaction of trial × paradigm on RTs and error rates, which reflects the observation that the switch costs were especially pronounced in the alternating runs compared to the voluntary task switching condition. This is consistent with results of authors like Liefooghe et al. (2010) who reported asymmetric switch costs in externally cued switching compared to the voluntary switching situations. In the study of Liefooghe et al. (2010), participants switched between the Stroop task conditions of reading and naming color and the authors found larger switch costs for the reading task in externally cued task switching only. As the existence of asymmetrical switch costs is often considered as an indicator for persisting activation of one of the tasks suppressing the processing of the other task (Allport et al., 1994), Liefooghe et al. interpreted the smaller expression of this asymmetry in voluntary task switching as evidence that the switch costs in this paradigm are more driven by active task reconfiguration processes. The pattern of our results is in line with the assumption that active control processes have started task reconfiguration in voluntary task switching earlier than the presentation time of the stimulus, which, in turn, could have decreased the switch costs in that condition. In the alternating runs paradigm, such earlier task reconfiguration is less possible (although not completely precluded), because the task cue is provided with the stimulus (Allport, et al., 1994; Arrington et al., 2010).

With regard to task selection in voluntary task switching, we did not observe effects of motivational salience and motivational state manipulation. Along with numerous task-switching studies, participants tended to repeat tasks more often than to switch between tasks, but the repetition bias was not significantly modulated by task or by hunger. We suggest that one reason for the absence of motivational modulation of task selection is the particular type of instruction that we used in the current study. Closely following instructions of Arrington and Logan (2004, 2005), our participants were asked to freely select tasks on a trial-by-trial basis with the constraint that both tasks had to be performed nearly equally often and in random order. A coin toss example used in the original instructions to illustrate the task decision process was left out. A possible direction for future study could be to relax instructions to better detect differences in task decisions for selecting motivationally salient vs. neutral task.

Finally, individual differences in restrictive eating as reflected by high values on the NIAS scale used to assess participants’ ARFID-like eating tendencies*,* were not associated with the ability to switch between tasks. We only found a non-significant trend towards a negative association between ARFID-like eating and switch costs when switching to the food task, whereas no association was found between ARFID-like eating and switch costs to the digit task. The reason for these results might be that our sample was too small to discover the significant effect using correlational analyses.

In addition, restrictive eating was measured by using a brief self-report questionnaire (NIAS), which might have over- or underestimated the level of restrictive eating (Vetrone et al., 2006). Therefore, future studies might apply more fine-grained measurements of the individual restrictive eating tendencies of participants in order to investigate the potential interaction between individual eating tendencies of participants and motivation-related food stimuli on the efficiency of cognitive control processes (Fairburn & Beglin, 1994; Vetrone et al., 2006).

## Summary

In sum, the current study indicates that motivation can modulate cognitive control in task switching when one of the tasks is motivationally salient. In its interaction with control processes, motivational salience varies with fluctuations in motivational states (hunger versus satiety). We suggest that depending on the salience of the tasks motivation accelerates or slows down processes of task set reconfiguration. Our research extends previous research (Paulitzki et al., 2008) by investigating interaction of affect, motivation and cognitive control in task switching in the context of eating behaviour further highlighting the need for inclusion of naturalistic manipulations of motivation in the motivation-control research.

## Data Availability

The data generated and/or analysed during the current study are available from the corresponding author on reasonable request.
